# Supplementary material for: In Silico Clinical Trials in Drug Development: A Systematic Review
Source: Ther Innov Regul Sci. 2025 Nov 24;60(2):423–39. doi: 10.1007/s43441-025-00893-w (PMC12945960; doi:10.1007/s43441-025-00893-w)
Supplement: Supplementary file 3 — Table S1 [file 43441_2025_893_MOESM3_ESM.pdf]

Table S1 List of the applications of registered trials in ClinicalTrials.gov for publications in PubMed

Author	Doi	Title	NCT number	Trial type	Purpose of gathered data	Abstract no.
Gutiérrez-Casares et al.	10.3389/fpsyt.2021.741170	Methods to Develop an in silico Clinical Trial: Computational Head-to-Head Comparison of Lisdexamfetamine and Methylphenidate	1>NCT00730249	interventional; Randomized; double blind; multiple arms	Model fitting for virtual population	17
			2>NCT00337285	interventional; Randomized; double blind; multiple arms	Model fitting for virtual population	
			3>NCT00763971	interventional; Non-Randomized; Open Label; single arm	Model fitting for virtual population	
Wang et al.	10.3389/fbioe.2020.00141	Conducting a Virtual Clinical Trial in HER2-Negative Breast Cancer Using a Quantitative Systems Pharmacology Model With an Epigenetic Modulator and Immune Checkpoint Inhibitors	1>NCT02453620	interventional; allocation: NA; Open Label; single arm	clinical evidence supporting	25
			2>NCT02115282	interventional; Randomized; double blind; multiple arms	used/ could be used to compare results	
Sové et al.	10.1136/jitc-2022-005414	Virtual clinical trials of anti-PD-1 and anti-CTLA-4 immunotherapy in advanced hepatocellular carcinoma using a quantitative systems pharmacology model	1>NCT01658878	interventional; Non-Randomized; Open Label; multiple arms	Model fitting for virtual population	56
Wang et al.	10.1136/jitc-2020-002100	Quantitative systems pharmacology model predictions for efficacy of atezolizumab and nab-paclitaxel in triple-negative breast cancer	1>NCT01375842	interventional; Randomized;Open Label/double blind; multiple arms	Model fitting for virtual population	81
			2>NCT02425891	interventional; Randomized;Open Label/double blind; multiple arms	clinical evidence supporting	
McClatchy et al.	10.1158/0008-5472.CAN-19-3883	Modeling Resistance and Recurrence Patterns of Combined Targeted-Chemoradiotherapy Predicts Benefit of Shorter Induction Period	1>NCT01553942	interventional; Randomized; Open Label; multiple arms	clinical evidence supporting	102
			2>NCT01822496	interventional; Randomized; Open Label; multiple arms	clinical evidence supporting	
			3>NCT00446225	interventional; Randomized; Open Label; multiple arms	model testing	
			4>NCT00686959	interventional; allocation: NA Open Label; single arm	model testing	
			5>NCT02415621	interventional; allocation: NA Open Label; single arm	clinical evidence supporting	

Table S1 ***Continued***

Author	Doi	Title	NCT number	Trial type	Purpose of gathered data	Abstract no.
Visentin et al.	10.1109/EMBC.2018.8513234	Long-acting Insulin in Diabetes Therapy: In Silico Clinical Trials with the UVA/Padova Type 1 Diabetes Simulator()	1>NCT01195454	interventional; Randomized; double blind; single arm	PKPD model fitting	104
			2>NCT01349855	interventional; Randomized; double blind; single arm	PKPD model fitting	
Jafarnejad et al.	10.1208/s12248-019-0350-x	A Computational Model of Neoadjuvant PD-1 Inhibition in Non-Small Cell Lung Cancer	1> NCT02259621	interventional; Non-Randomized; Open Label; multiple arms	Model fitting for virtual population	107
Jenner et al.	10.1136/jitc-2020-001387	In silico trials predict that combination strategies for enhancing vesicular stomatitis oncolytic virus are determined by tumor aggressivity	1>NCT02285816	interventional; Non-Randomized; Open Label; multiple arms	used/ could be used to compare results	119
			2>NCT02879760	interventional; Non-Randomized; Open Label; multiple arms	used/ could be used to compare results	
			3>NCT03865212	interventional; allocation: NA; Open Label; single arm	used/ could be used to compare results	
Visentin et al.	10.1089/dia.2016.0128	Improving Efficacy of Inhaled Technosphere Insulin (Afrezza) by Postmeal Dosing: In-silico Clinical Trial with the University of Virginia/Padova Type 1 Diabetes Simulator	1>NCT0144595	study not found	clinical evidence supporting	116
			2>NCT01544881	interventional; Randomized; Open Label; multiple arms	PKPD model fitting	
			3>NCT01445951	interventional; Randomized; Open Label; multiple arms	reference for model building (dosage)	
Gong et al.	10.3390/cancers13153751	A Spatial Quantitative Systems Pharmacology Platform spQSP-IO for Simulations of Tumor-Immune Interactions and Effects of Checkpoint Inhibitor Immunotherapy	1>NCT02259621	interventional; Non-Randomized; Open Label; multiple arms	reference for model building	154
Dickschen et al.	10.1186/2193-1801-3-285	Concomitant use of tamoxifen and endoxifen in postmenopausal early breast cancer: prediction of plasma levels by physiologically-based pharmacokinetic modeling	1>NCT01273168	interventional; allocation: NA/Non-Randomized;	medical evidence supporting	188
			2>NCT01327781	Open Label; single arm	medical evidence supporting	

Table S1 ***Continued***

Author	Doi	Title	NCT number	Trial type	Purpose of gathered data	Abstract no.
Milberg et al.	10.1038/s41598-019-47802-4	A QSP Model for Predicting Clinical Responses to Monotherapy, Combination and Sequential Therapy Following CTLA-4, PD-1, and PD-L1 Checkpoint Blockade	1>NCT00730639	interventional; Non-Randomized; Open Label; single arm	PKPD model fitting	197
			2>NCT00324155	interventional; Non-Randomized; Open Label; single arm	used/ could be used for comparing results	
			3>NCT01024231	interventional; Randomized; double blind; multiple arms	used/ could be used for comparing results	
			4>NCT00729664	interventional; Non-Randomized; Open Label; muliple arms	used/ could be used for comparing results	
			5>NCT01783938	interventional; Randomized; Open Label; muliple arms	used/ could be used for comparing results	
Mazzocco et al.	10.1155/2015/297903	Increasing the Time Interval between PCV Chemotherapy Cycles as a Strategy to Improve Duration of Response in Low-Grade Gliomas: Results from a Model-Based Clinical Trial Simulation	1> NCT01967095	interventional; allocation: NA; Open Label; single arm	model informs trial design	208
Zhang et al.	10.1101/2023.08.11.553000	Informing virtual clinical trials of hepatocellular carcinoma with spatial multi-omics analysis of a human neoadjuvant immunotherapy clinical trial	1>NCT03299946	interventional; allocation: NA; Open Label; single arm	model testing	236
Qi et al.	10.1002/psp4.12896	Virtual clinical trials: A tool for predicting patients who may benefit from treatment beyond progression with pembrolizumab in non-small cell lung cancer	1>NCT00540514	interventional; Randomized; Open Label; multiple arms	model fitting for virtual population	245
Gong et al.	10.3389/fphar.2023.1163432	Using quantitative systems pharmacology modeling to optimize combination therapy of anti-PD-L1 checkpoint inhibitor and T cell engager	1>NCT02259621	interventional; Non-Randomized; Open Label; single/ multiple arms	Model fitting for virtual population	255
			2>NCT02650713	interventional; Non-Randomized; Open Label; single/ multiple arms	Model fitting for virtual population	
			3>NCT02788279	interventional; Non-Randomized; Open Label; single/ multiple arms	Model fitting for virtual population	
Cheng et al.	10.1158/2767-9764.CRC-23-0257	Mathematical Modeling Identifies Optimum Palbociclib-fulvestrant Dose Administration Schedules for the Treatment of Patients with Estrogen Receptor-positive Breast Cancer	1> NCT02630693	interventional; Non-Randomized; Open Label; single arm	used/could be used for comparing model rsults	291
			2> NCT04557449	interventional; Randomized; Open Label; multiple arms	clinical evidence supporting	

The examples were chosen according to the registered clinical trial in ClinicalTrials.gov. The table depicts the NCT number, the trial types, and the purpose of gathered data considered in the publication. Abstract number refers to Supplementary material.
