# Supplementary material for: In Silico Clinical Trials in Drug Development: A Systematic Review
Source: Ther Innov Regul Sci. 2025 Nov 24;60(2):423–39. doi: 10.1007/s43441-025-00893-w (PMC12945960; doi:10.1007/s43441-025-00893-w)
Supplement: Supplementary file 4 — Table S2 [file 43441_2025_893_MOESM4_ESM.pdf]

Table S2 **List of the publications in PubMed linked to the registered Trials in ClinicalTrials.gov**

NCT number	URL	Title of registered trial	PubMed number	Title of publication	Doi	Abstract no.
NCT00879658	https://ClinicalTrials.gov/show/NCT00879658	Safety, Tolerability, Efficacy and Optimal Dose Finding Study of BAF312 in Patients With Relapsing-remitting Multiple Sclerosis	1>26083135	Characterization of dose-response for count data using a generalized MCP-Mod approach in an adaptive dose-ranging trial	10.1002/pst.1693	56
			2>23764350	Siponimod for patients with relapsing-remitting multiple sclerosis (BOLD): an adaptive, dose-ranging, randomised, phase 2 study	10.1016/S1474-4422(13)70102-9	
NCT05833802	https://clinicaltrials.gov/study/NCT05833802	Computation Prediction of Drug Response Based on Omics Data	1>31561483	The Need for Multi-Omics Biomarker Signatures in Precision Medicine	10.3390/ijms20194781	78
			2>34157485	DAGM: A novel modelling framework to assess the risk of HER2-negative breast cancer based on germline rare coding mutations	10.1016/j.ebiom.2021.103446	
NCT05716854.	https://clinicaltrials.gov/study/NCT05716854	Electrophysiological Effects of Potential QT Prolonging Drugs	1>28878692	Optimization of an In silico Cardiac Cell Model for Proarrhythmia Risk Assessment	10.3389/fphys.2017.00616	92
			2>30151907	Assessment of an In Silico Mechanistic Model for Proarrhythmia Risk Prediction Under the CiPA Initiative	10.1002/cpt.1184	
			3>29209226	Uncertainty Quantification Reveals the Importance of Data Variability and Experimental Design Considerations for in Silico Proarrhythmia Risk Assessment	10.3389/fphys.2017.00917	
			4>28202629	Improving the In Silico Assessment of Proarrhythmia Risk by Combining hERG (Human Ether-à-go-go-Related Gene) Channel-Drug Binding Kinetics and Multichannel Pharmacology	10.1161/CIRCEP.116.004628	

The examples were chosen according to the publications in PubMed listed in the according clinical trial. The table depicts the NCT number, the title of trials and the title of publications. Abstract number refers to Supplementary material.
