# Supplementary material for: In Silico Clinical Trials in Drug Development: A Systematic Review
Source: Ther Innov Regul Sci. 2025 Nov 24;60(2):423–39. doi: 10.1007/s43441-025-00893-w (PMC12945960; doi:10.1007/s43441-025-00893-w)
Supplement: Supplementary file 5 — Figures_Supplementary [file 43441_2025_893_MOESM5_ESM.pdf]

Supplmentary Figure

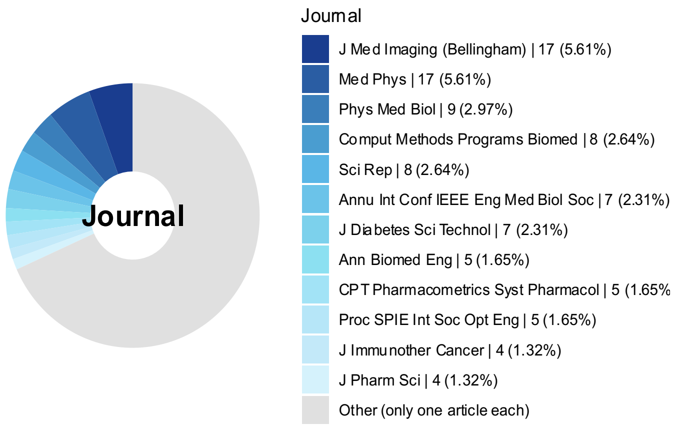

Figure S1. The pie chart illustrates the distribution of journal information for all reviewed articles from PubMed, with darker blue indicating higher usage and gray representing categories with journals accounting for less than 1%.

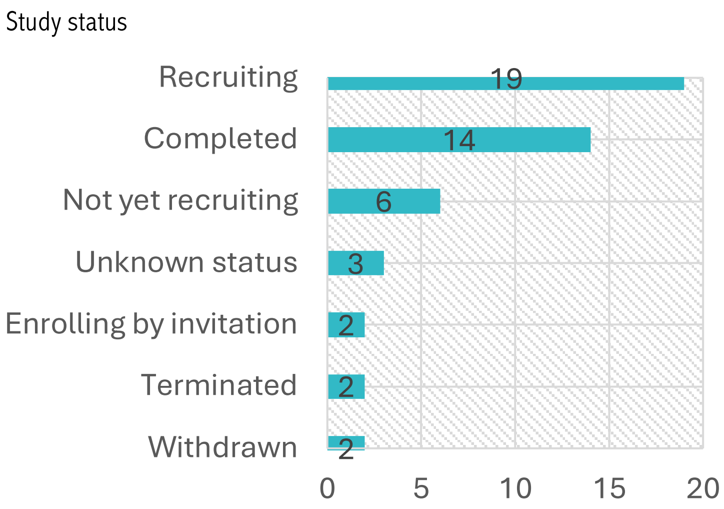

Figure S2. The bar chart illustrates the distribution of study status from ClinicalTrials.gov.

Study results

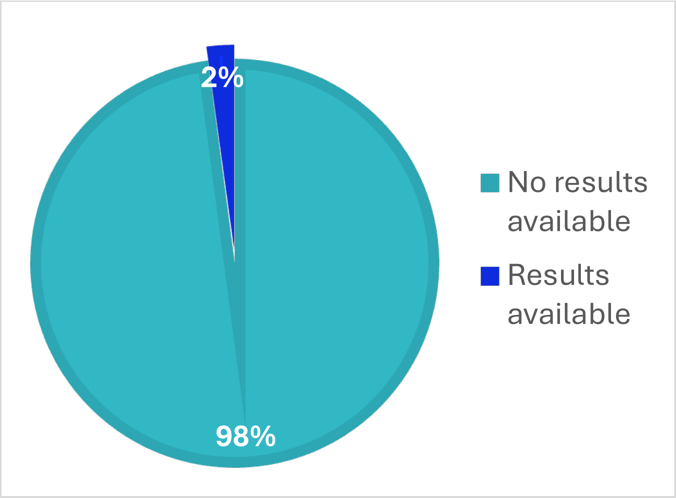

Figure S3. The pie chart illustrates the distribution of study results from ClinicalTrials.gov.
