# Supplementary material for: In Silico Clinical Trials in Drug Development: A Systematic Review
Source: Ther Innov Regul Sci. 2025 Nov 24;60(2):423–39. doi: 10.1007/s43441-025-00893-w (PMC12945960; doi:10.1007/s43441-025-00893-w)
Supplement: Supplementary file 6 — Categories_Supplementary [file 43441_2025_893_MOESM6_ESM.docx]

Categories used for systematic review

The following tables contain a description of the categories used to classify the paper found in PubMed (PM) and clinical trials found in clinicaltrials.gov (Cl). The definition of the categories differ between the two data sources.

1. In silico (IS) connection (PM)

| IS review | - Summary of current and/ or past research - Class of diseases and/ or methods |
| --- | --- |
| IS application | - Holistic view of a trial - Testing new drug, drug combination or dose - Specific disease - Focus is on work **with the** model |
| IS methods | - Describing only part of an IS trial - Introducing new model or method for conducting an IS trial - Exemplary application on diseases/ treatments - Focus is on the **model** **itself** |

1. In silico (IS) connection (Cl)

| IS application | - Using simulations as a substantial part of the trial (e.g. dose finding, virtual twin to facilitate treatment decision) |
| --- | --- |
| preparation for future IS trials | - Studies with the purpose of collecting data e.g. to determine specific parameter values with the mentioned aim to facilitate in-silico trials |
| validation of IS trial results | - Referencing a specific in-silico trial - Same/ similar study design as for in-silico trial |
| none | - Using AI to analyse images - Using results derived from in-silico trials in minor parts of the study |

1. Diseases (PM and Cl)

**Classification using the following 28 Chapters of the ICD-11 system from the WHO (**[**https://icd.who.int/en**](https://icd.who.int/en)**):**

01 Certain infectious or parasitic diseases

02 Neoplasms

03 Diseases of the blood or blood-forming organs

04 Diseases of the immune system

05 Endocrine, nutritional or metabolic diseases

06 Mental, behavioural or neurodevelopmental disorders

07 Sleep-wake disorders

08 Diseases of the nervous system

09 Diseases of the visual system

10 Diseases of the ear or mastoid process

11 Diseases of the circulatory system

12 Diseases of the respiratory system

13 Diseases of the digestive system

14 Diseases of the skin

15 Diseases of the musculoskeletal system or connective tissue

16 Diseases of the genitourinary system

17 Conditions related to sexual health

18 Pregnancy, childbirth or the puerperium

19 Certain conditions originating in the perinatal period

20 Developmental anomalies

21 Symptoms, signs or clinical findings, not elsewhere classified

22 Injury, poisoning or certain other consequences of external causes

23 External causes of morbidity or mortality

24 Factors influencing health status or contact with health services (used for papers describing imaging especially cancer screening or other diagnostic procedures done without a given diagnosis beforehand)

25 Codes for special purposes

26 Supplementary Chapter Traditional Medicine Conditions

V Supplementary section for functioning assessment

X Extension Codes (used for papers regarding medication without disease specification, e.g. analgesics)

**And a new category:**

General diseases (used mainly for review papers or method papers introducing models describing a whole system e.g. immune or hormone system)

1. Previously developed model (PM)

Note: In some cases the use of a previously described model still necessitates calibration and validation.

| Yes | - Commercial simulation platform used (e.g. Simcyp, Padova) - Specific model was derived/ described in a previously published paper |
| --- | --- |
| no | - Extending previously developed models with new functionalities (e.g. new compartments) - Using a model in a novel context, so that in particular validation becomes necessary |

1. Source of data for calibration and validation of the models (PM)

| Pre-clinical data | - In-vitro data - In-vivo data - Genetic data |
| --- | --- |
| Clinical data | - Data from a clinical trial or observational study - Imaging data - Pharmacokinetic and pharmacodynamic data |
| Registries | - Databases like health records - demographic data |
| Mechanistic knowledge | - values of parameters already known from literature (from former trials or studies) - sets of ODEs or PDEs for (partially) describing a biological process - fully developed and ready to use models are kept track of in the column *previously developed model*, so **no extra** mechanistic knowledge is needed |
| Simulation data | - data derived from simulation studies based on other models |

1. Relevance to drug therapy development (PM and Cl)

| specified | - Testing dosing regimens and/or predicting drug (adverse-) effects - Context of developing new drug or drug therapy (e.g. repurposing existing drugs, determine new combination) |
| --- | --- |
| unspecified | - Evaluating the overall impact of in silico on drug development e.g. review paper - Developing new models of biological systems with regard to future drug development (among other things) |
| no | - Imaging trials - Reviews that don’t specify their use in drug development - Model building for surgical methods evaluation |

1. Rare disease (PM and Cl)

| yes | - Existing entry in the Orpha.net - Prevalence of disease in population described in the paper is less than 1/2000 |
| --- | --- |
| no | - Diseases with rare subtypes (e.g. certain types of lung cancers) when the paper is only about the broad group (e.g. lung cancer) |

1. Pediatric diseases (PM and Cl)

| yes | - Disease is only or mostly prevalent in people younger than 18 years e.g. gliomas |
| --- | --- |
| no | - E.g. ADHD as it occurs not considerably less in adults (4.5%) than in children (5.6-7.6%) |
